# Arrythmogenic cardiomyopathy. Let's have a closer look to the left ventricle. Report of our experience

**DOI:** 10.1186/1532-429X-17-S1-Q74

**Published:** 2015-02-03

**Authors:** Eloisa Feliu, Rafal Moscicki, Vicente Climent, Emilio Galcerá-Jornet, Amaya Garcia Fernandez, Alejandro Pascual

**Affiliations:** 1 MR Unit. Inscanner S.L., Hospital General Universitario de Alicante, Alicante, Spain; 2 Cardiology, Hospital General Universitario de Alicante, Alicante, Spain

## Background

The classic Arrythmogenic Cardiomyopathy (ARC) has predilection for the right ventricle (RV). However, recent studies have demonstrated another pattern with early and predominant left ventricular involvement, Left Dominant Arrythmogenic Cardiomyopathy (LDAC). In our centre the alarm went off when CMR studies in relatives of sudden death (SD) patients with autopsy proven ARC, demonstrated predominant LV involvement. These findings together with the literature, encouraged us to look for more cases with this particular pattern among our patients.

Our objective was to study clinical presentation of patients with CMR pattern of LDAC and try to find any prognostic parameters of clinical events.

## Methods

With the finding of a diffuse mesocardial and subepicardial Late Gadolinium Enhancement (LGE) in SD patient's relatives as well as 3 patients with SD, we decided to review retrospectively the CMR and clinical database of our centre to obtain more cases with this pattern compatible with LDAC. We also included prospectively some more cases among patients scheduled for CMR during 2013-2014.

## Results

We found 33 patients (median age 56 years, 76% males) with images suggestive of LDAC. The main indications for a CMR study were arrhythmias (43%), Dilated Cardiomyopathy (DCM)(21%), family history of SD (15%), chest pain with normal coronary arteries and myocarditis (12%). Four patients, studied because of family history of SD, had LV myocardial LGE and were asymptomatic. Midwall and/or subepicardial pattern of LGE (100%), fatty epicardial infiltration (66.7%) and LV segmental contractility abnormalities (59%) were the most common findings. Echocardiography was normal in most of the cases.

Thirteen patients presented VT and/or SD. In 9 cases ICD implantation was indicated. Autopsy performed in one case of SD was positive for LDAC, and the genetic study found a mutation of FLNC gene responsible for filamin synthesis.

We have found that fatty infiltration (p= 0.022) and not severity of LGE (p= 0.46) was associated with the development of adverse clinical events.

## Conclusions

Until the quite recent widespread use of CMR, LDAC was an under-recognized entity. Meso-subepicardial hyperenhancement was recently described as one of the image criteria of LDAC. We found this pattern in patients with clinically suspected DCM, chest pain with normal coronary arteries or myocarditis, as well as in asymptomatic patients. However, its severity was not found to be related to development of clinical events. On the contrary, fatty infiltration was more frequently found in patients with VT and/or SD. Since the form of presentation can be very variable, and echocardiography can be normal, we should have it mind, because of its possible bad evolution.

## Funding

No fundings nor conflicts of interest.

**Figure 1:**
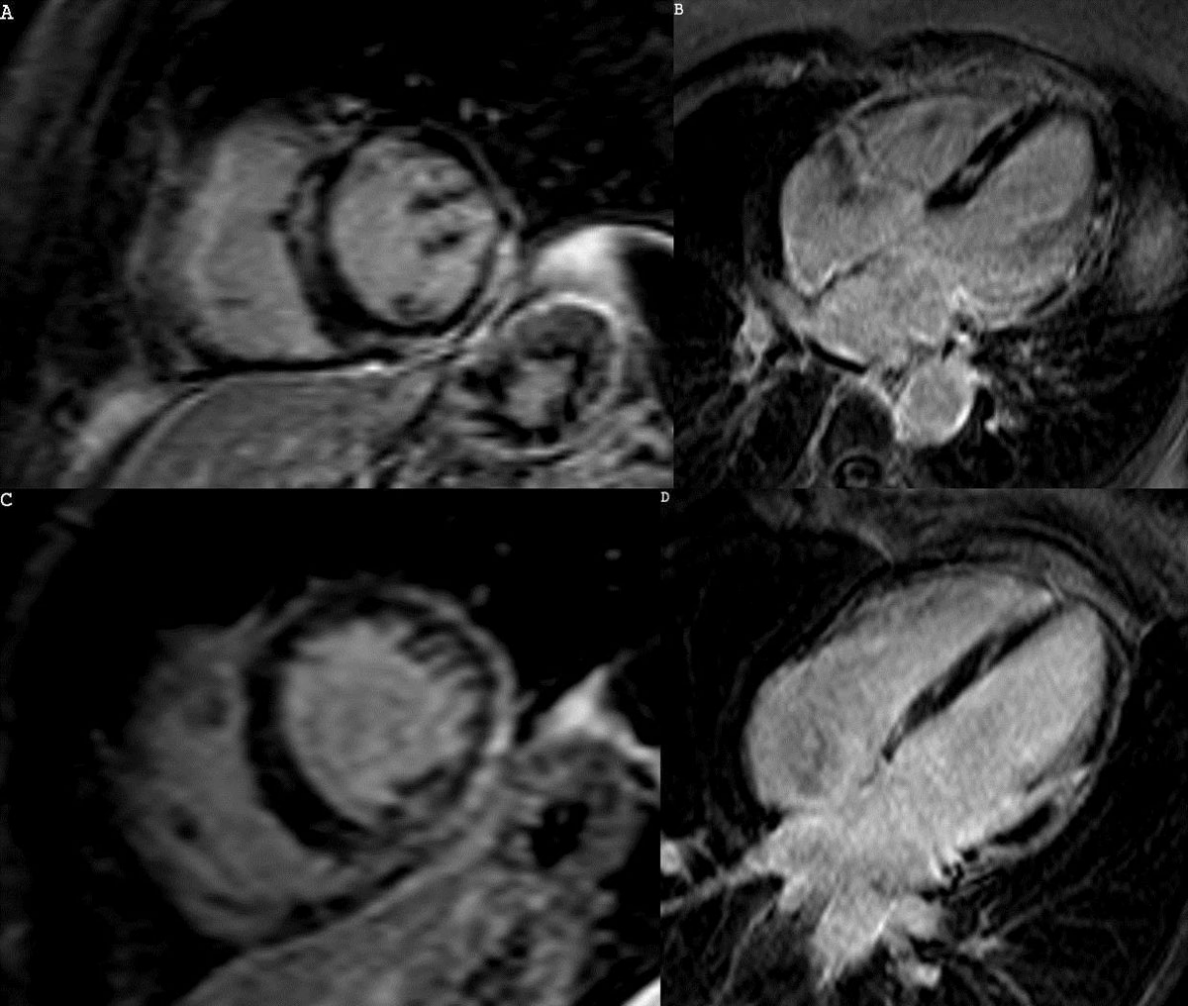
LGE images of an asymptomatic mother (A-B) and brother (C-D) of a SD patient with autopsy proven LDAC. Both of them show septal mesocardial hyperenhancement and inferior and lateral wall meso-subepicardial hyperenhancement

**Figure 2:**
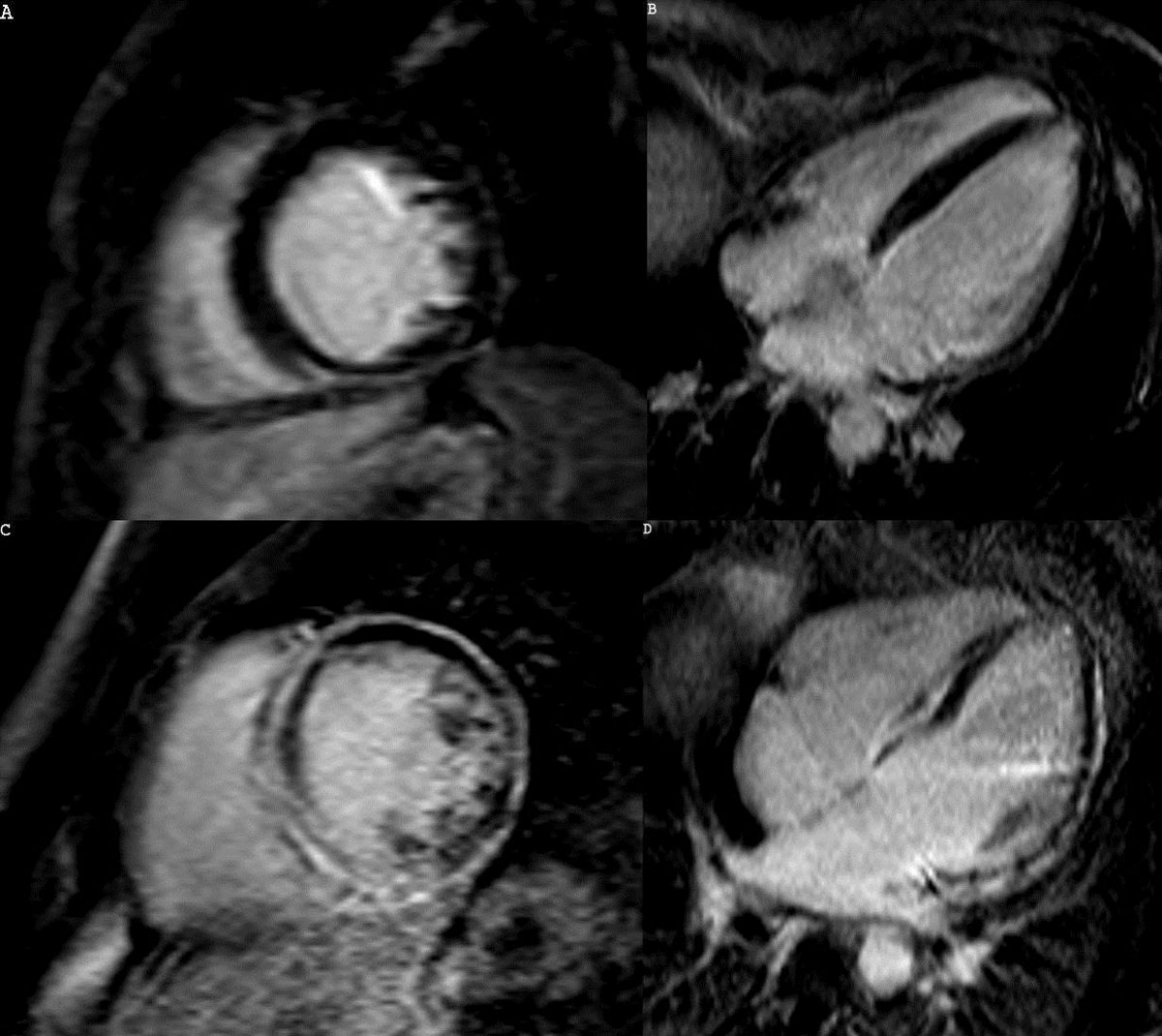
LGE images of an aborted SD patient (A-B) and a SD patient (C-D). Only inferior wall mild subepicardial hyperenhancement is seen in the first patient while diffuse meso-subepicardial hyperenhancement is seen in the second, the latter also with autopsy proven LDAC.

